# Development and validation of a nomogram to predict synchronous lung metastases in patients with ovarian cancer: a large cohort study

**DOI:** 10.1042/BSR20203089

**Published:** 2020-11-24

**Authors:** Yufei Yuan, Fanfan Guo, Ruoran Wang, Yidan Zhang, Guiqin Bai

**Affiliations:** 1 Medicine Department, Xi’an Jiaotong University, Xi’an, China; 2 Department of Critical Care Medicine, West China Hospital of Sichuan University, Sichuan, China; 3 Department of Obstetrics and Gynecology, The First Affiliated Hospital of Xi’an Jiaotong University, Xi’an, China

**Keywords:** Mitochondrial dysfunction, Mitochondria unfolded protein response (mtUPR), monitoring epidemiology final results, nomogram, receiver operating characteristic curve

## Abstract

Purpose: Lung metastasis is an independent risk factor affecting the prognosis of ovarian cancer patients. We developed and validated a nomogram to predict the risk of synchronous lung metastases in newly diagnosed ovarian cancer patients.

Methods: Data of ovarian cancer patients from the Surveillance, Epidemiology, and Final Results (SEER) database between 2010 and 2015 were retrospectively collected. The model nomogram was built on the basis of logistic regression. The consistency index (C-index) was used to evaluate the discernment of the synchronous lung metastasis nomogram. Calibration plots were drawn to analyze the consistency between the observed probability and predicted probability of synchronous lung metastases. The Kaplan–Meier method was used to estimate overall survival rate, and influencing factors were included in multivariate Cox regression analysis (*P*<0.05) to determine the independent prognostic factors of synchronous lung metastases.

Results: Overall, 16059 eligible patients were randomly divided into training (*n*=11242) and validation cohorts (*n*=4817). AJCC T, N stage, bone metastases, brain metastases, and liver metastases were evaluated as predictors of synchronous lung metastases. Finally, a nomogram was constructed. The nomogram based on independent predictors was calibrated and showed good discriminative ability. Mixed histological types, chemotherapy, and primary site surgery were factors affecting the overall survival of patients with synchronous lung metastases.

Conclusion: The clinical prediction model has high accuracy and can be used to predict lung metastasis risk in newly diagnosed ovarian cancer patients, which can guide the treatment of patients with synchronous lung metastases.

## Introduction

Ovarian cancer is among the most common malignant tumors in the female reproductive system. Ovarian cancer is the fifth most common cause of cancer-related deaths among American women. In 2018, an estimated 14070 people died of ovarian cancer in the United States [1]. Since the symptoms of ovarian cancer are unclear and there is currently no effective screening method, most patients are already at advanced stages (III and IV) at the time of diagnosis, accompanied by synchronous distant metastases [2,3].

Lung metastasis is the third most common distant metastatic site of ovarian cancer, accounting for 28.42% of distant metastatic sites. The location of distant metastases is an independent prognostic factor for overall survival [4]. Previous studies show that the risk factors for distant metastases are stage, grade, and lymph node involvement [5]. However, the sample size of the study was small. There are few studies on the risk factors of synchronous lung metastases, and most of them are case reports [6,7]. The median interval between the diagnosis of ovarian cancer and recording of metastatic disease was 44 months [5].

Identifying the risk factors for synchronous lung metastases can ensure that high-risk patients are thoroughly investigated at the initial diagnosis.These patients can then be treated as early as possible or provided with appropriate preventive treatment. A large number of studies and realistic evidence is also needed to determine the risk factors for synchronous lung metastases in ovarian cancer patients.

The purpose of the present study was to use Surveillance, Epidemiology, and End Results (SEER) database to characterize the prevalence, related factors, and prognostic factors of synchronous lung metastases in ovarian cancer patients. At the same time, a nomogram to predict the risk of synchronous lung metastases was developed on the basis of clinical factors, which may guide screening.

## Methods

### Study population

**Figure 1:**
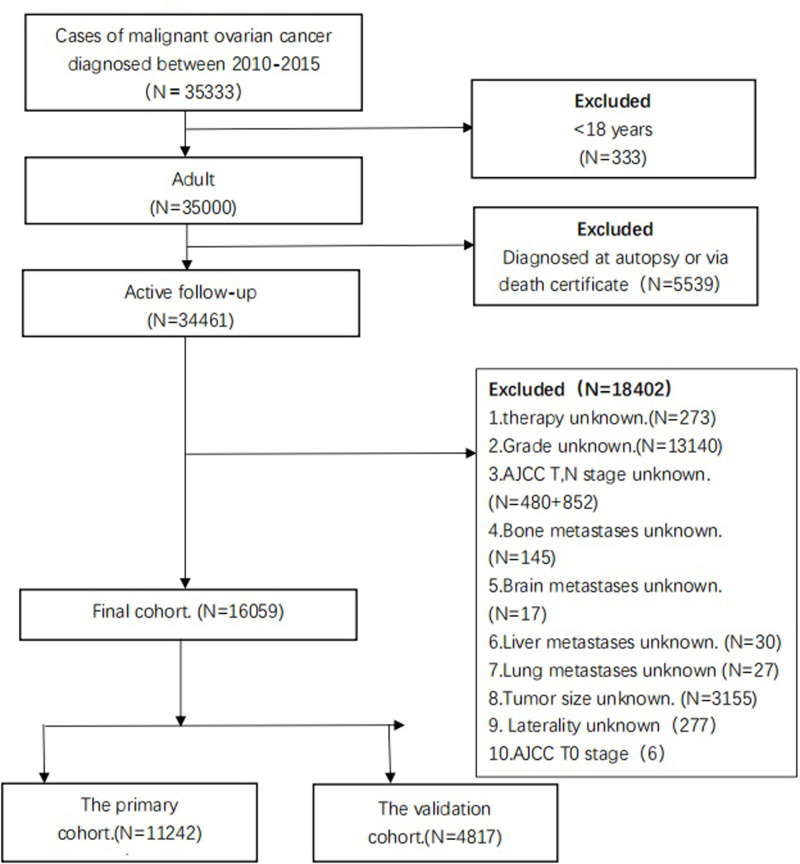
Flowchart of patients’ selection

Data were obtained from the SEER database. The SEER *Stat 8.3.5 software (https://seer.cancer.gov/data/) was used to access the database. The site code was restricted to the ovary. Since the details of metastases were not recorded before 2010, patients with primary cancer of the ovary, aged ≥ 18 years at diagnosis, between 2010 and 2015 were analyzed. The exclusion criteria for patient selection included the following: (1) unknown grade; (2) unknown AJCC T, N stage and AJCC T0 stage; (3) unknown metastases information; (4) unknown tumor size; (5) unknown laterality; and (6) unknown therapy information. The flowchart of the subjects’ selection is listed in Figure 1. According to the inclusion and exclusion criteria, 16059 patients with ovarian cancer were finally enrolled in our study. We further randomly divided the patients in a 7:3 ratio to form a training cohort (*n*=11242) for nomogram construction and a validation cohort (*n*=4817) for internal verification.

Data regarding clinical characteristics including age, race, marital status, insurance status, year of diagnosis, household income at diagnosis, histological type, grade, laterality, clinical AJCC T, N stage, tumor size, metastatic status, and therapy information were collected from the SEER database. Since all information from the SEER database was identified and no personal identifying information was used in this analysis, informed consent was not required. The present study complied with the 1964 Helsinki Declaration, its later amendments, and comparable ethical standards.

### Statistical analysis

Statistical analysis was performed using the SPSS 21 software. Categorical data were presented as frequency (%) and analyzed using the chi-squared test. The Kolmogorov–Smirnov test was used to verify the normality of variables. Normally distributed variables were expressed as mean ± standard deviation, while non-normally distributed variables were expressed as median (interquartile range). Hazard ratios and 95% confidence intervals (CIs) were calculated. Univariate and multivariate logistic regression analyses were used to determine the risk factors of synchronous lung metastases in patients with ovarian cancer. Factors with a *P*-value less than 0.05 were incorporated into the multivariable logistic regression model.

A synchronous lung metastases nomogram was formulated on the basis of the results of multivariate logistic analysis using the rms package in R version 3.6.1 (R Foundation for Statistical Computing, Vienna, Austria; www.r-project.org). Receiver operating characteristic (ROC) curves were drawn. Finally, we evaluated the stability of the prognostic nomogram and the synchronous lung metastasis nomogram by internal validation with 1000 bootstrap samples. The nomograms were validated both internally and externally. The C-index (Harrell’s concordance index) was used to assess the exact predicted values of nomograms. Calibration plots were drawn to analyze the consistency between the observed and predicted probabilities. Overall survival was estimated by the Kaplan–Meier method, and the difference between distinct groups was compared using the log-rank test. A multivariable Cox regression model, incorporating the significant factors in the Kaplan–Meier method (*P*<0.05) was conducted to analyze the independent prognostic factors for synchronous lung metastases.

## Results

### Patients’ basic information

According to the inclusion and exclusion criteria, data of 16059 of the 35333 ovarian cancer patients registered between 2010 and 2015 were collected from the SEER database. The patients were divided into training (*n*=11242) and verification (*n*=4817) groups. The basic information of the patients is listed in Table 1. The median age of the patients was 59 years. Among these patients, 13223 (82.3%) were white, 1057 (6.6%) were black, and 1711 (10.7%) were of other races. A total of 3377 (21.0%) patients were unmarried, 8549 (53.2%) were married, and 3486 (21.7%) were separated. The number of insured and uninsured patients was 861 (3.5%) and 15337 (95.5%), respectively. The median household income was 6255. The number of patients with tumor diameters <2 cm, 2–5 cm, >5 cm was 1311 (8.1%), 2678 (16.7%), and 12076 (75.2%), respectively. A total of 4947 (30.8%) patients had tumors on the left, 5109 (31.8%) patients with tumors on the right, and 6003 (37.4%) patients with tumors on both sides. The number of well differentiated, moderately differentiated, poorly differentiated, and undifferentiated histology tumors was 2011 (12.5%), 2758 (17.2%), 6395 (39.8%), and 4895 (30.5%), respectively. The number of T1, T2, and T3 stage tumors was 5500 (34.2%), 2552 (15.9%), and 8007 (49.9%), respectively. The numbers of N0 and N1 stages were 12514 (77.9%) and 3545 (22.1%), respectively. Bone metastases occurred in 54 (0.3%), brain metastases in 15 (0.1%), liver metastases in 572 (3.6%), and lung metastases in 411 (2.6%) patients. The number of histology-type serous, endometrioid, mucinous, clear cell, carcinosarcoma, malignant Brenner, carcinoma, NOS, mixed, and other was 8644 (53.8%), 2367 (14.7%), 1071 (6.7%), 1124 (7.0%), 515 (3.2%), 18 (0.1%), 516 (3.2%), 1140 (7.1%), and 664 (4.1%), respectively. The chi-square test for all variables between the two groups yielded *P*>0.05.

**Table 1:** Demographical and clinical characteristics between patient with the training cohort and validation cohort

**Variables**	The training cohort (*n*=11242)		The validation cohort (*n*=4817)		Total (*n*=16059)		*P*-value
	Number	%	Number	%	Number	%	
**Age**	59	59	59	0.360			
**Race**							0.750
White	9267	82.4	3956	82.1	13223	82.3	
Black	725	6.4	332	6.9	1057	6.6	
Other (American Indian/AK Native, Asian/Pacific Islander)	1201	10.7	510	10.6	1711	10.7	
Unknown	49	0.4	19	0.4	68	0.4	
**Marital status**							0.363
Unmarried	2329	20.7	1049	21.8	3377	21.0	
Married	5987	53.3	2562	53.2	8549	53.2	
Separated	2473	22.0	1013	21.0	3486	21.7	
Unknown	453	4.0	194	4.0	647	4.0	
**Insurance status**							0.577
Uninsured	403	3.6	158	3.3	561	3.5	
Insured	10724	95.4	4613	95.8	15337	95.5	
Unknown	115	1.0	46	1.0	161	1.0	
**Household income**	6204 (5716–8008)	6325 (5716–8008)	6255 (5716–8008)	0.394			
**Year of diagnosis**							0.210
2010	1783	15.9	755	15.7	2539	15.8	
2011	1850	16.5	806	16.7	2656	16.5	
2012	1825	16.2	816	16.9	2641	16.4	
2013	1873	16.7	825	17.1	2698	16.8	
2014	1951	17.4	759	15.8	2710	16.9	
2015	19610	17.4	856	17.8	2816	17.5	
**Tumor size**							0.892
<2 cm	906	8.1	399	8.3	1311	8.1	
2–5 cm	1875	16.7	803	16.7	2678	16.7	
>5 cm	8461	75.3	3615	75.0	12076	75.2	
**Laterality**							0.628
Left	3471	30.9	1476	30.6	4947	30.8	
Right	35965	32.0	1514	31.4	5109	31.8	
Bilateral	4176	37.1	18287	37.9	6003	37.4	
**Grade**							0.426
Well differentiated	1417	12.6	594	12.3	2011	12.5	
Moderately differentiated	1904	16.9	854	17.7	2758	17.2	
Poorly differentiated	4460	39.7	1935	40.2	6395	39.8	
Undifferentiated	34621	30.8	1434	29.8	4895	30.5	
**AJCC T stage**							0.805
T1	3835	34.1	1665	34.6	5500	34.2	
T2	1783	15.9	769	16.0	2552	15.9	
T3	5624	50.0	2383	49.5	8007	49.9	
**AJCC N stage**							0.497
N0	8747	77.8	37710	78.3	12514	77.9	
N1	2498	22.2	1047	21.7	3545	22.1	
**Bone metastasis**							0.592
No	1120	99.7	4799	99.6	16005	99.7	
Yes	36	0.3	18	0.4	54	0.3	
**Brain metastasis**							0.159
No	11229	99.9	4815	100.0	16044	99.9	
Yes	13	0.1	2	0.0	15	0.1	
**Liver metastasis**							0.681
No	10846	96.5	4641	96.3	15487	96.4	
Yes	396	3.5	176	3.7	572	3.6	
**Lung metastasis**							0.681
No	10959	97.5	4689	97.3	15648	97.4	
Yes	283	2.5	128	2.7	411	2.6	
**Histological type**							0.866
Serous	6016	53.5	2628	54.6	8644	53.8	
Endometrioid	1662	14.8	705	14.6	2367	14.7	
Mucinous	758	6.7	313	6.5	1071	6.7	
Clear cell	779	6.9	345	7.2	1124	7.0	
Carcinosarcoma	361	3.2	154	3.2	515	3.2	
Malignant Brenner	14	0.1	4	0.1	18	0.1	
Carcinoma, NOS	362	3.2	154	3.2	516	3.2	
Mixed	813	7.2	327	6.8	1140	7.1	
Other	477	4.2	187	3.9	664	4.1	
**Surgery (primary)**							0.292
No	149	72.0	58	70.0	207	70.0	
Yes	11093	28.0	4759	30.0	15852	30.0	
**Radiation**							0.493
No	11090	70.0	4751	69.7	15841	70.0	
Yes	152	30.0	66	30.0	218	30.0	
**Chemotherapy**							0.841
No	2753	70.1	1172	70.0	3925	70.0	
Yes	8489	29.9	3645	30.0	12134	30.0	

### Risk factors for lung metastasis

Univariable logistic analysis showed that factors closely related to the occurrence of lung metastasis included the following: older patient age (OR = 1.015; 95% CI, 1.006–1.025; *P*=0.001), bilateral tumors (OR = 1.556; 95% CI, 1.179–2.053; *P*=0.002), lower differentiation grade (poorly differentiated OR = 5.288; 95% CI, 2.583–10.825; *P*≤0.001; undifferentiated OR = 6.435; 95% CI, 3.139–13.195; *P*≤0.001), higher AJCC T stage (T2 OR = 4.991; 95% CI, 2.859–8.712; *P*≤0.001; T3 OR = 8.796; 95% CI, 5.432–14.243; *P*<0.001), higher AJCC stage N (OR = 2.863; 95% CI, 2.254–3.635; *P*<0.001), bone (OR = 15.403; 95% CI, 7.355–32.256; *P*<0.001), brain (OR = 17.443; 95% CI, 5.340–56.981; *P*<0.001), liver metastases (OR = 10.483; 95% CI, 7.822–14.050; *P*<0.001), and mucinous (OR = 0.425; 95% CI, 0.190–0.953; *P*=0.038) and clear cell histological subtypes (OR = 0.248; 95% CI, 0.077–0.794; *P*=0.019).

Multivariable logistic regression analysis showed that higher T and N stages, and the presence of bone, liver, and brain metastases were associated with the earlier development of synchronous lung metastases (Table 2).

**Table 2:** Univariable and multivariable logistic regression for analyzing the associated factors for developing lung metastases in training cohort

**Variables**	Univariable			Multivariable		
	OR	95% Cl	*P*-value	OR	95% Cl	*P*-value
**Age**	1.015	1.006–1.025	**0.001**	1.010	0.999–1.021	0.086
**Race**			0.622			0.114
White	References			References		
Black	1.193	0.758–1.878	0.445	1.174	0.725–1.899	0.515
Other	1.236	0.865–1.767	0.244	1.595	1.089–2.336	0.016
Unknown	0.000	0 .000	0.998	0.000	0.000	0.997
**Marital status**			0.287			0.144
Unmarried	References			References		
Married	1.034	0.761–1.404	0.832	0.868	0.626–1.203	0.396
Separated	1.074	0.751–1.535	0.697	0.770	0.520–1.139	0.191
Unknown	0.437	0.174–1.096	0.078	0.352	0.136–0.912	0.032
**Insurance status**			0.772			0.418
Uninsured	References			References		
Insured	0.913	0.496–1.683	0.772	0.730	0.378–1.411	0.349
Others/Unknown	1.284	0.401–4.111	0.674	1.221	0.357–4.174	0.750
**Household income**	1.000	1.000–1.000	0.794	1.000	1.000–1.000	0.622
**Year of diagnosis**			0.666			0.742
2010	References			References		
2011	1.293	0.835–2.001	0.250	1.283	0.817–2.015	0.278
2012	1.367	0.886–2.109	0.158	1.389	0.889–2.172	0.149
2013	1.140	0.729–1.784	0.565	1.166	0.736–1.847	0.51
2014	1.381	0.902–2.117	0.138	1.348	0.865–2.100	0.187
2015	1.323	0.860–2.033	0.202	1.197	0.765–1.873	0.432
**Tumor size**			0.080			0.207
<2 cm	References			References		
2–5 cm	1.291	0.717–1.822	0.303	1.026	0.620–1.696	0.921
>5 cm	0.925	0.541–1.240	0.726	0.796	0.505–1.255	0.326
**Laterality**			**<0.001**			0.082
Left	References			References		
Right	0.704	0.500–0.991	0.044	0.666	0.467–0.951	0.025
Bilateral	1.556	1.179–2.053	0.002	0.840	0.620–1.138	0.261
**Grade**			**<0.001**		0.000	0.246
Well differentiated	References			References		
Moderately differentiated	2.154	0.960–4.829	0.063	1.355	0.592–3.101	0.471
Poorly differentiated	5.288	2.583–10.825	<0.001	1.590	0.736–3.437	0.238
Undifferentiated	6.435	3.139–13.195	<0.001	1.890	0.868–4.118	0.109
**AJCC T stage**			**<0.001**	References		**<0.001**
T1	References					
T2	4.991	2.859–8.712	<0.001	3.032	1.675–5.485	<0.001
T3	8.796	5.432–14.243	<0.001	4.055	2.343–7.019	<0.001
**AJCC N stage**			**<0.001**			**<0.001**
N0	References			References		
N1	2.863	2.254–3.635	<0.001	1.696	1.313–2.190	<0.001
**Bone metastasis**			<0.001			**<0.001**
No	References			References		
Yes	15.403	7.355–32.256	<0.001	5.945	2.373–14.894	<0.001
**Brain metastasis**			**<0.001**			**<0.001**
No	References			References		
Yes	17.443	5.340–56.981	<0.001	13.375	3.449–51.865	<0.001
**Liver metastasis**			<0.001	<0.001		<0.001
No	References			References		
Yes	10.483	7.822–14.050	<0.001	6.292	4.605–8.598	<0.001
**Histological type**			<0.001			0.503
Serous	References			References		
Endometrioid	1.581	0.832–3.005	0.162	0.807	0.449–1.450	0.473
Mucinous	0.425	0.190–0.953	0.038	0.526	0.180–1.535	0.240
Clear cell	0.248	0.077–0.794	0.019	0.536	0.240–1.195	0.127
Carcinosarcoma	0.423	0.160–1.120	0.083	1.513	0.879–2.605	0.135
Malignant Brenner	2.166	0.971–4.831	0.059	0.000	0.000	0.999
Carcinoma, NOS	0.000	0.000	0.999	1.184	0.659–2.125	0.572
Mixed	2.160	0.968–4.817	0.060	1.052	0.632–1.751	0.844
Other	1.057	0.484–2.310	0.889	1.172	0.547–2.512	0.682

Bold values indicate statistical significance (*P*<0.05).

### Nomogram development

**Figure 2:**
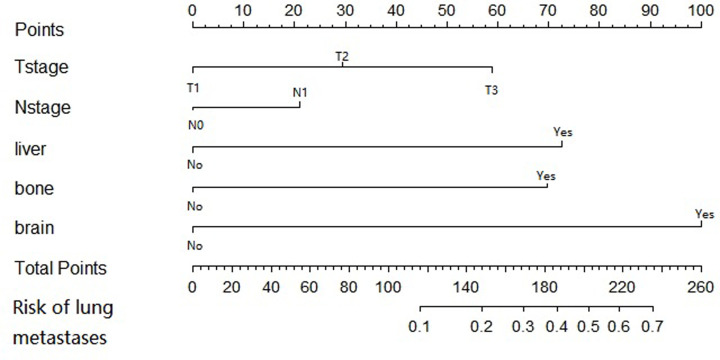
Nomogram for predicting synchronous lung metastases in ovarian cancer patients A synchronous lung metastases nomogram was formulated on the basis of the results of multivariable logistic analysis using the rms package in R version 3.6.1. The first line shows the point assignment of each variable. Lines 2–6 indicate the variables included in the nomogram. For individual patients, each variable is assigned a point value based on tumor characteristics. The points assigned to each of the five variables are added, and the total points are displayed in the seventh line. The bottom row shows the possibility of synchronous lung metastases.

A nomogram to predict synchronous lung metastases in patients with ovarian cancer was developed in the training cohort. The risk factors determined by multivariable logistic regression analysis, including higher T and N stage, and the development of bone, liver, and brain metastases were developed and used as the final nomogram (Figure 2).

### ROC curves analysis and prediction value evaluation

**Figure 3:**
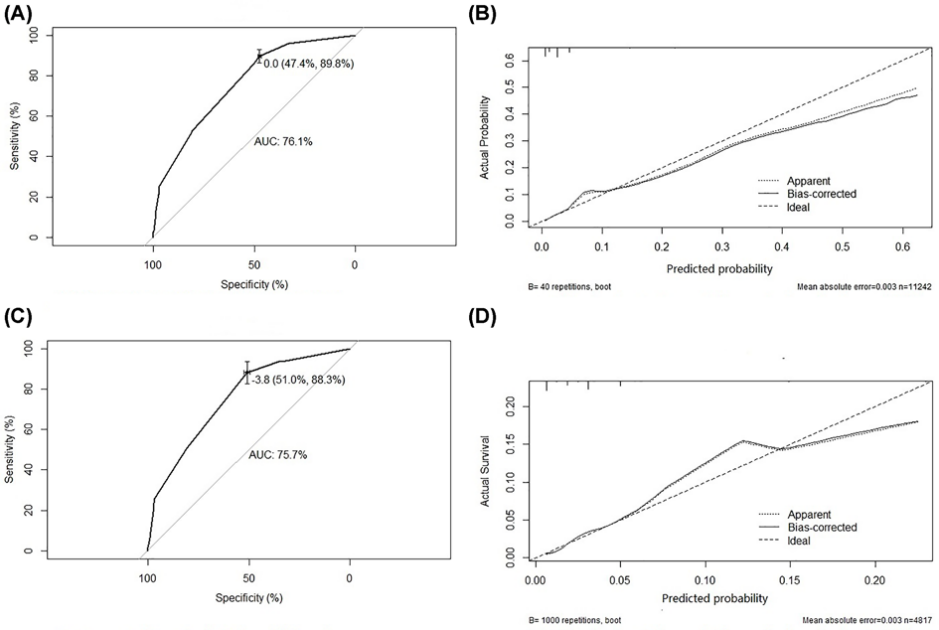
Identification and calibration of the nomogram in the training and verification cohorts (**A**) ROC curve for discrimination in the training cohorts. (**B**) Calibration plots for the actual (observed) and predicted probabilities of the nomograms in the training cohorts. (**C**) ROC curve for discrimination in the validation cohorts. (**D**) Calibration plots for the actual (observed) and predicted probabilities of the nomograms in the validation cohorts. Using the rms package in R version 3.6.1, the ROC curve and calibration diagram were drawn. (A,C) ROC curve for discrimination in the training and validation cohorts. (B,D) Calibration plots for the actual (observed) and predicted probabilities of the nomograms in the training and verification cohorts. The x-axis represents the predicted probability of the nomogram measured by logistic regression analysis, and the y-axis represents the actual probability. The vertical line represents the frequency distribution of the predicted probabilities. The dashed line represents the ideal reference line, where the predicted probability matches the observed probability. Calibration plots showed excellent calibration of the nomogram.

ROC curves were drawn to determine the predicted value of the nomogram of synchronous lung metastases in the training and validation cohorts. As shown in Figure 3A,C, ROC curves were drawn. We verified the nomogram internally and externally. The C-index was used to evaluate the prediction accuracy of the nomogram. As shown in Figure 3B, the internal verification of the nomogram was performed, and the C-index was 0.761 (0.736–0.787). As shown in Figure 3D, the external verification of the validation cohort showed that the C index was 0.757 (−0.718 to 0.795). Verification of the nomogram showed agreement with the predicted values.

### Survival analysis and prognostic factors of synchronous lung metastases

**Figure 4:**
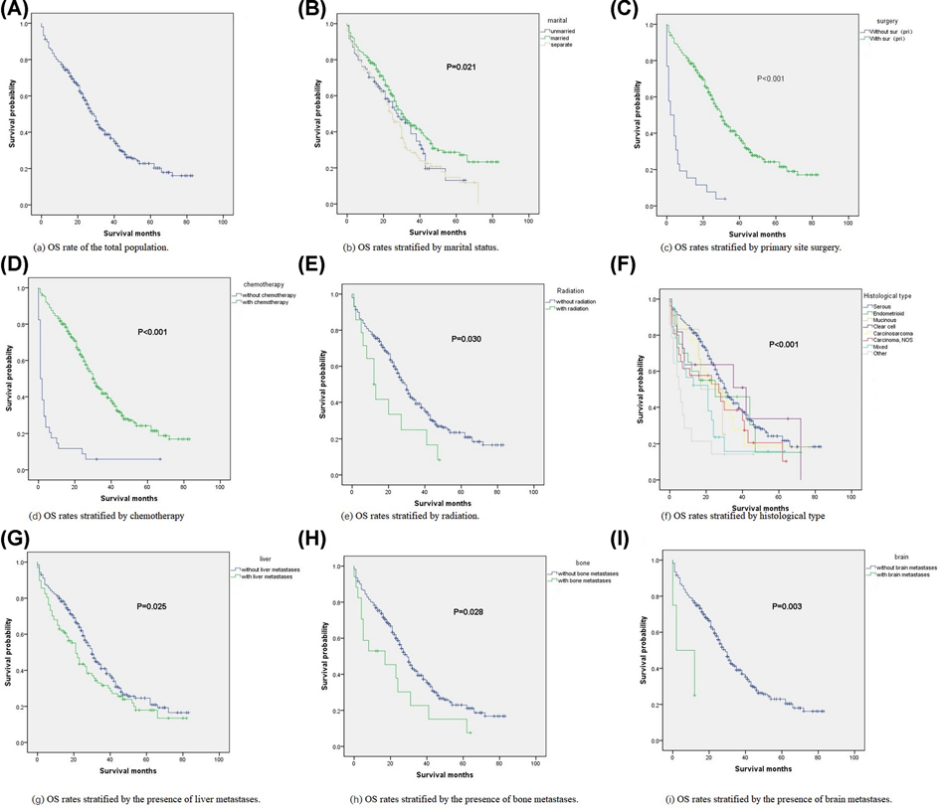
Kaplan–Meier analysis of the overall survival of ovarian cancer patients with lung metastasis The overall survival (OS) rate was estimated by the Kaplan–Meier method, and the log-rank test was used to compare the differences between different groups. (**A**) OS rate of the total population. (**B**) OS rates stratified by marital status. (**C**) OS rates stratified by primary site surgery. (**D**) OS rates stratified by chemotherapy. (**E**) OS rates stratified by radiation. (**F**) OS rates stratified by histological type. (**G**) OS rates stratified by the presence of liver metastases. (**H**) OS rates stratified by the presence of bone metastases. (**I**) OS rates stratified by the presence of brain metastases. Multivariate Cox regression results incorporating the above important factors showed that mixed histological type (hazard ratio [HR] = 2.531; 95% CI: 1.538–4.165; *P*<0.001) was positively correlated with overall mortality. Primary site surgery (HR = 0.315; 95% CI: 0.190–0.522; *P*<0.001) and chemotherapy (HR = 0.216; 95% CI: 0.139–0.335; *P*<0.001) were beneficial for survival (Table 3).

The 3- and 5-year overall survival rates of ovarian cancer patients were 72.2 and 58.1%, respectively. For the 411 patients with newly diagnosed lung metastases, the 3- and 5-year survival rates were 33.8 and 22.8%, respectively (Figure 4A). Kaplan–Meier analysis showed that the overall survival of married patients (Figure 4B, *P*=0.021), primary site surgery (Figure 4C, *P*<0.01), chemotherapy (Figure 4D, *P*<0.01), and radiation (Figure 4E, *P*=0.030) were higher than those of the control group. Mixed histological type (Figure 4F, *P*<0.001), liver metastases (Figure 4G, *P*=0.025), bone metastases (Figure 4H, *P*=0.028), and brain metastases (Figure 4I, *P*=0.003) correlated negatively with overall survival rate. Kaplan–Meier analysis was used to estimate the overall survival rate. The influencing factors selected by the Kaplan–Meier method were included in the multivariate Cox regression (*P*<0.05) to analyze the independent prognostic factors of synchronous lung metastases. Mixed histological types (*P*<0.001), chemotherapy (*P*<0.001), and primary site surgery (*P*<0.001) affected the overall survival of ovarian cancer patients with synchronous lung metastases (Table 3).

**Table 3:** Multivariable Cox regression for analyzing the associated factors for prognostic factors patients with lung metastases

**Variables**	Multivariable		
	OR	95% Cl	*P*-value
**Histological type**			**0.003**
Serous	References		
Endometrioid	1.391	0.764–2.532	0.280
Mucinous	1.081	0.391–2.990	0.880
Clear cell	1.106	0.515–2.374	0.797
Carcinosarcoma	1.457	0.854–2.488	0.167
Carcinoma, NOS	0.981	0.575–1.673	0.942
Mixed	2.531	1.538–4.165	<0.001
Other	2.585	1.362–4.908	0.004
**Surgery (primary)**			**<0.001**
No	Reference		
Yes	0.315	0.190–0.522	<0.001
**Chemotherapy**			**<0.001**
No	Reference		
Yes	0.216	0.139–0.335	**<0.001**

Bold values indicate statistical significance (*P*<0.05).

## Discussion

Ovarian cancer is the seventh most common cancer among women and the eighth most common cause of cancer death worldwide, with a 5-year overall survival rate of <50% [8]. Two-thirds of the patients are already at advanced stages at the time of diagnosis (Stage III/IV) [9]. When the lungs are affected, the main route of metastasis is through the pleura. Lung metastases usually represent as visceral pleura involvement and continuous infiltration. Occasionally, isolated lesions are observed. Invasion of lymphatic and blood vessels also occurs [10]. The incubation period from the diagnosis of ovarian cancer to the development of lung metastases can be as long as 108 months [11]. Compared with standard chemotherapy treatment alone, early detection of lung metastases can increase the chances of timely, more aggressive treatments, which may lead to prolonged survival [4]. Active chemotherapy can significantly reduce the tumor load and metastasis of ovarian cancer [12]. Surgical removal of isolated lung metastatic lesions is reasonable [13]. Targeted therapy is also a promising treatment for metastatic ovarian cancer [14]. Routine imaging studies, such as computed tomography or magnetic resonance imaging, have not shown high sensitivity and specificity when diagnosing micrometastases <1 cm [15]. Therefore, there is a need for a non-invasive method to predict the likelihood of synchronous lung metastases in ovarian cancer patients. We used data from the SEER database to develop and validate the predicted nomogram, which demonstrated significant discernment and calibration capabilities and can provide a personalized estimation of the likelihood of synchronous lung metastases in ovarian cancer patients.

To the best of our knowledge, the present study is the first to generate a risk model based on clinical and tumor characteristics through population-based surveillance, epidemiology, and final result databases to predict the risk of synchronous lung metastases in newly diagnosed ovarian cancer patients. We found that the higher the AJCC T and N stages, the higher the likelihood of metastases which is similar to likelihood of bone metastasis of ovarian cancer and the findings of other types of tumor metastases research [16–18]. Previous studies have shown that poor differentiation and lymph node involvement are risk factors for distant metastasis [4]. We found that liver metastases, brain metastases, and bone metastases are risk factors for synchronous lung metastases. If distant metastases are found in other parts of the body, it means that the cancer has metastasized [19], and the probability of lung metastases is higher.

We verified the nomogram internally and externally. The nomogram of synchronous lung metastases includes five factors: AJCC T stage, AJCC N stage, bone metastases, liver metastases, and brain metastases. The nomogram showed agreement between the predicted results and the observed results in the verification. In addition, the C-indices of internal verification and external verification of the nomogram were 0.761 (0.736–0.787) and 0.757 (0.718–0.795), respectively, indicating consistency with the predicted values. For patients with a higher risk of synchronous metastases predicted by this model, imaging examination should be performed on time to diagnose the occurrence of lung metastases in the initial period, so as to better guide clinical procedures.

The determination of prognostic factors related to synchronous lung metastases in these patients may help doctors to provide targeted treatment strategies for patients at different risk levels and improve patient survival and quality of life. Previous studies have shown that lung metastases can significantly worsen the prognosis of patients [20]. The median survival time for the diagnosis of distant disease is 12 months [5]. In this study, the 3- and 5-year survival rates for 411 patients with synchronous lung metastases were 33.8 and 22.8%, respectively, similar to other studies [21,22]. Primary site surgical treatment and chemotherapy can improve overall survival. Therefore, for patients with ovarian cancer with synchronous lung metastases, active surgery, and chemotherapy are encouraged. At the same time, the mixed histological type is a high-risk factor for mortality, and physicians should attach great importance to it. The present study has several limitations that should be noted. The main limitation is that the variables used to construct the nomogram only used clinico-pathological features because there were no important tumor biomarkers in the SEER database. Another limitation is that although the established nomogram shows good discrimination and verification capabilities, it still requires further verification based on large-scale external queues. Third, only patients with synchronous lung metastases were analyzed. Since they may not be recorded in the SEER databases, metachronous lung metastases that occurred later in the disease were not analyzed. This was a retrospective study. The patients were selected from the hospital, so there was a selection bias.

## Conclusion

Lung metastasis is an independent risk factor affecting the prognosis of patients with ovarian cancer. In the first diagnosis of ovarian cancer, early detection of synchronous lung metastases through routine screening is beneficial for high-risk patients.

The present study is the first to use population-based SEER database to generate a risk model based on clinical and tumor characteristics to predict the risk of synchronous lung metastases in newly diagnosed ovarian cancer patients with high accuracy. The present study preliminarily determined the prognostic factors related to synchronous lung metastases in patients with ovarian cancer, which will help doctors to provide targeted treatment strategies for patients at different risk levels and improve the survival rate and quality of life of patients.

## Data Availability

https://seer.cancer.gov/data/ is available for the Surveillance, Epidemiology, and End Results Program database.

## Abbreviations

AJCC: American Joint Committee on Cancer
CI: confidence interval
C-index: consistency index/Harrell’s concordance index
OR: odds ratio
ROC: receiver operating characteristic
SEER: Surveillance, Epidemiology, and Final Results
